# Preferential Impairment of the Contralesional Posterior Semicircular Canal in Internuclear Ophthalmoplegia

**DOI:** 10.3389/fneur.2017.00502

**Published:** 2017-09-22

**Authors:** Seung-Han Lee, Sang-Hoon Kim, Sung-Sik Kim, Kyung Wook Kang, Alexander Andrea Tarnutzer

**Affiliations:** ^1^Department of Neurology, Chonnam National University Hospital, Chonnam National University, Gwangju, South Korea; ^2^Department of Neurology, Chonnam National University Medical School, Chonnam National University, Gwangju, South Korea; ^3^Department of Neurology, University Hospital Zurich, University of Zurich, Zurich, Switzerland

**Keywords:** vestibulo-ocular reflex, internuclear ophthalmoplegia, medial longitudinal fasciculus, head impulse test, compensatory saccade

## Abstract

**Background:**

The vertical vestibulo-ocular reflex (VOR) may be impaired in internuclear ophthalmoplegia (INO) as the medial longitudinal fasciculus (MLF) conveys VOR-signals from the vertical semicircular canals. It has been proposed that signals from the contralesional posterior semicircular canal (PSC) are exclusively transmitted through the MLF, while for the contralesional anterior canal other pathways exist.

**Objective:**

Here, we aimed to characterize dysfunction in individual canals in INO-patients using the video-head-impulse test (vHIT) and to test the hypothesis of dissociated vertical canal impairment in INO.

**Methods:**

Video-head-impulse testing and magnetic resonance imaging were obtained in 21 consecutive patients with unilateral (*n* = 16) or bilateral (*n* = 5) INO and 42 controls. VOR-gains and compensatory catch-up saccades were analyzed and the overall function (normal vs. impaired) of each semicircular canal was rated.

**Results:**

In unilateral INO, largest VOR-gain reductions were noted in the contralesional PSC (0.55 ± 0.11 vs. 0.89 ± 0.08, *p* < 0.001), while in bilateral INO both posterior (0.43 ± 0.11 vs. 0.89 ± 0.08, *p* < 0.001) and anterior (0.58 ± 0.19 vs. 0.88 ± 0.09, *p* < 0.001) canals showed marked drops. Small, but significant VOR-gain reductions were also found in the other canals in unilateral and bilateral INO-patients. Impairment of overall canal function was restricted to the contralesional posterior canal in 60% of unilateral INO-patients, while isolated involvement of the posterior canal was rare in bilateral INO-patients (20%). Reviewers correctly identified the INO-pattern in 15/21 (71%) patients and in all controls (sensitivity = 84.2% [95%-CI = 0.59.5–95.8]; specificity = 95.5% [95%-CI = 83.3–99.2]).

**Conclusion:**

Using a vHIT based overall rating of canal function, the correct INO-pattern could be identified with high accuracy. The predominant and often selective impairment of the contralesional posterior canal in unilateral INO further supports the role of the MLF in transmitting posterior canal signals. In patients with acute dizziness and abnormal vHIT-results, central pathologies such as INO should be considered as well, especially when the posterior canal is involved.

## Introduction

One of the most common neuro-ophthalmologic syndromes that result from medial longitudinal fasciculus (MLF) damage is internuclear ophthalmoplegia (INO). It is characterized by slowing of/or limited adduction during horizontal eye movements in the affected eye and dissociated abducting nystagmus in the intact eye (1–3). Besides these cardinal deficits, the vertical vestibulo-ocular reflex (VOR) can be impaired in INO since the MLF conveys VOR-signals from the vertical semicircular canals and the otolith organs (4, 5). INO is often accompanied by a contraversive ocular tilt reaction (OTR) that is largely due to interruption of the graviceptive pathways from the contralateral utricle or the contralateral vertical semicircular canals, which ascend in the MLF after decussation in the lower pons (6, 7). Dissociated vertical-torsional nystagmus (jerky seesaw nystagmus) may be present and is explained by disruption of the neural pathways from the contralateral vertical semicircular canals with or without concomitant damage of the fibers from the contralateral utricle (5, 8).

The head-impulse test (HIT), first described in 1988 (9), is a critical component in the bedside assessment of the integrity of the VOR. It can be used to evaluate the function of each semicircular canal individually (10). In specialty practice, the horizontal HIT is now widely used to distinguish between peripheral- and central-vestibular disorders (11, 12). The recently developed video-head-impulse test (vHIT) is a useful tool to quantify dysfunction in the horizontal and vertical semicircular canals (13, 14). These non-invasive video-oculography (VOG) devices usually consist of lightweight goggles, similar in appearance to swimming goggles, with an embedded high-speed (often ≥ 250 frames/s) infrared video camera(s) to track eye movements and inertial accelerometers in the frame to measure head movements (15).

The aim of this study was to confirm and further quantify previously reported VOR deficits and neuro-opthalmologic findings in a larger group of patients with INO [as confirmed by neurologic examination and magnetic resonance imaging (MRI)] using non-invasive recording techniques (vHIT). Based on anatomical considerations and previous observations in a single patient with unilateral INO using invasive measurement techniques (scleral search coils) (16), we predicted significantly reduced function of the contralesional posterior semicircular canal (PSC) in our patients with unilateral INO compared to healthy controls. Previously, mild to moderate impairment was noted also for the contralesional anterior semicircular canal (ASC), which has led to the hypothesis that signals from the ASC are not exclusively transmitted by the MLF (16). We will assess whether this observation holds true for a larger group of subjects as well. In analogy, for patients with bilateral INO, bilateral involvement of both the PSCs and ASCs are predicted. We will also assess the diagnostic accuracy of the vHIT in INO compared to MR-imaging and clinical examination, providing sensitivity and specificity values.

## Materials and Methods

### Patient Selection

Between March 2014 and December 2016, we contacted all potential study participants presenting to the emergency department or the outpatient clinic of the Department of Neurology, Chonnam National University Hospital, Gwangju, South Korea. Twenty-five consecutive patients were eligible. Four patients had to be excluded because of incomplete data (missing vHIT, *n* = 2) or revision of diagnosis (one-and-a-half syndrome, *n* = 2). Eventually, 21 patients with unilateral (*n* = 16) or bilateral (*n* = 5) INO with typical neurologic (i.e., dissociated nystagmus of the abducting eye and slowing or palsy of the adducting eye) and radiologic [brainstem lesions including the MLF on T2, fluid-attenuated inversion recovery (FLAIR) or diffusion-weighted imaging (DWI) on MRI] findings were enrolled. The OTR including skew deviation, head tilt and ocular torsion (OT) was examined at the bedside and by fundus photography. VOG (SLMed, Seoul, Korea) was performed in sitting position for the detection of spontaneous nystagmus (i.e., dissociated vertical-torsional nystagmus, downbeat nystagmus, upbeat nystagmus).

A total of 42 healthy control subjects were enrolled (21 females, 21 males, mean age = 58.9 years; range: 21–83 years). All subjects received a detailed neurologic examination and video-head-impulse testing and did not show evidence of central or peripheral vestibulopathy.

The study design was thoroughly explained to all participants. This study was carried out in accordance with the recommendations of the Institutional Review Board of the Chonnam National University Hospital (Gwangju, South Korea) with written informed consent from all subjects. All subjects gave written informed consent in accordance with the Declaration of Helsinki. The protocol was approved by the Institutional Review Board of the Chonnam National University Hospital (Gwangju, South Korea).

### Video-Head-Impulse Testing

Patients underwent a structured bedside neuro-otologic examination in the emergency department or in the dizziness clinic of the Chonnam National University Hospital. We captured HIT measurements using a lightweight, portable VOG device (ICS Impulse; GN Otometrics, Taastrup, Denmark). Patients were seated and asked to fixate a distant target (~1.5 m away). Eye position was calibrated using laser targets projected from the goggles. After calibration, the examiner performed a series of horizontal HITs toward each ear. Vertical HITs were applied to all subjects along the left-anterior-right-posterior and right-anterior-left-posterior canal plane (13). Target head velocity was between 150 and 200°/s and head displacement ranged between 10° and 20°. The pre-specified target number of HITs per canal was 20.

OtosuiteV 4.0 (GN Otometrics) was used for gain-analysis of the vHIT recordings. The VOR-gain was calculated as the ratio of cumulative slow-phase eye velocity over cumulative head velocity from the onset of the head impulse to the moment when head velocity returned to 0 (13). Each compensatory saccade was visualized to ensure accurate characterization. Overt saccades occurred in the opposite direction of the head rotation and reached peak acceleration after the head had stopped moving. Covert saccades occurred in the opposite direction of the head rotation and reached peak acceleration before the head had stopped moving (17). Traces with artifacts (e.g., blinks during the HIT) were removed interactively (18). If spontaneous nystagmus was present, OtosuiteV 4.0 was operated in the nystagmus-adjusted interpretation mode, which alters filtering algorithms for determining inadequate impulses and calculates VOR-gain measures by accounting for the spontaneous, slow-phase drift of the eye.

All vHIT traces were independently reviewed by two neuro-otologists with extensive experience (AAT, SHL) without knowledge of the clinical findings and the results from MR-imaging. The manufacturer of the video-goggles used (GN Otometrics) proposed cutoff values in VOR-gain for the horizontal (0.8) and the vertical (0.7) canals, which were used here. Noteworthy, these values were in agreement with normative values for a wide range of ages reported (15). Traces were evaluated by the reviewers for reduced VOR-gain, increased corrective saccades or a combination of both (14). Inter-rater agreement for individual canal function (normal vs. pathological) in all 63 subjects included (21 patients and 42 controls) was 0.89 (Cohen’s kappa) (19). Based on the individual canal ratings, the reviewers also provided a rating of the suspected underlying diagnosis (left-sided INO, right-sided INO, bilateral INO, healthy control). Note that for this rating the reviewers knew that subjects were either controls or suffered from INO. For a diagnosis of unilateral INO, impairment of the contralateral PSC was required, whereas for bilateral INO impairment of both PSCs was mandatory. Discordant ratings were resolved by discussion among the two reviewers.

In addition, the frequency of compensatory saccades was assessed by the same two neuro-otologists (SHL, AAT) and assigned to one of three grades as follows: grade 0 = no compensatory saccades at all; grade 1 = compensatory saccades in less than 50% of head-impulses for a given canal and subject; grade 2 = compensatory saccades observed in more than 50% of head-impulses in a given canal and subject. Note that this rating was qualitative and did not consider a specific cutoff amplitude for single saccades.

### Neuroimaging

According to the imaging protocol of the Chonnam National University Hospital, patients suspected to have acute stroke underwent MR-imaging at the emergency department immediately after hospital admission. The MRI protocol consisted of DWI, FLAIR, gradient-echo imaging, and time-of-flight MR angiography in a sequential manner. In case of suspected demyelinating disease, gadolinium-enhanced T1-weighted imaging was also performed. MR-sequences were analyzed independently by two neurologists (Lee Seung-Han and Kim Sung-Sik) who were blinded to the clinical data. Discrepancies were resolved by consensus.

### Statistics

For both patients and controls, distribution of data of each canal was assessed for normality using the Jarque-Bera hypothesis test of composite normality (jbtest.m, Matlab 7.0). As our data were normally distributed, mean ± 1 SD values were provided when pooling individual data points. Statistical analysis was performed using SPSS 23 (IBM, Armonk, NY, USA). We applied a generalized linear model for unilateral and bilateral INO patients vs. controls, and main effects included the group unilateral INO: *n* = 3 (ipsilesional, contralesional, controls); bilateral INO: *n* = 2 (patients, controls); and the canals (*n* = 3). The level of significance was kept at *p* = 0.05, and Fisher’s least significant difference method was used to correct for multiple comparisons.

## Results

### Demographics and Neuro-Ophthalmologic Findings

**Figure 1:**
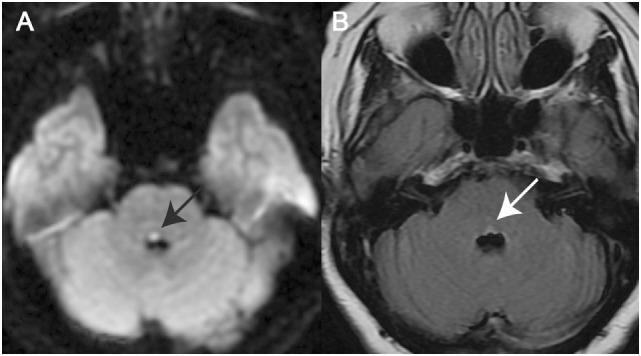
Magnetic resonance imaging (MRI) in two illustrative cases with internuclear ophthalmoplegia (INO). **(A)** Axial diffusion-weighted imaging–MRI in a patient with unilateral INO (patient 7) demonstrating a right-sided paramedian dorsal pontine lesion (black arrow). **(B)** Axial fluid-attenuated inversion recovery-image in a patient with bilateral INO (patient 19) showing a hyperintense bilateral paramedian dorsal pontine lesion (white arrow).

A total of 21 patients (8 females, 13 males) with a mean age of 62.8 years (SD = 17.0 years) were enrolled. In this study, all patients showed a unilateral or bilateral medial pontine tegmental lesion (see Figure 1 for two examples). The underlying cause of INO was either ischemic stroke (*n* = 17) or a demyelinating disorder (*n* = 4) (Table 1).

**Table 1:** Clinical characteristics of 21 patients with internuclear ophthalmoplegia.

No.	Age range (years)	Side of INO	Etiology	Sx-to-VOG (days)	SN (initial)	Sx-to-HIT (days)	OTR
1	41–45	R	Demyelination	3	U/D, CW	7	L
2	71–75	R	Infarct	37	U, CW	29	L (OT)
3	46–50	R	Infarct	3	None	3	L (OT)
4	46–50	R	Infarct	5	U, CW	5	L (OT)
5	76–80	R	Infarct	7	None	10	L (OT)
6	46–50	R	Infarct	8	None	8	L (OT)
7	51–55	R	Infarct	1	U, CW	105	L-OT
8	71–75	R	Infarct	5	None	628	L-OT
9	66–70	R	Infarct	5	None	760	L-OT
10	71–75	R	Infarct	3	U/D, CW	1,072	L-OT
11	51–55	L	Infarct	1	U, CCW	1	R
12	71–75	L	Infarct	1	U, CCW	2	R-OT
13	71–75	L	Infarct	2	None	2	R-OT
14	41–45	L	Infarct	2	U, CCW	5	R-OT
15	91–95	L	Infarct	11	None	45	R-OT
16	76–80	L	Infarct	NA	NA	234	R-OT
17	19–20	B	Demyelination	5	D	5	None
18	16–20	B	Demyelination	131	U	131	None
19	41–45	B	Demyelination	20	D	20	None
20	66–70	B	Infarct	15	None	117	None
21	51–55	B	Infarct	3	None	752	None

*B, bilateral; CCW, counter-clockwise torsional; CW, clockwise torsional; D, downbeat; F, female; INO, internculear ophthalmoplegia; L, left; Lat, lateralization; M, male; NA, not available; OT, ocular torsion; OTR, ocular tilt reaction; R, right; Sx-to-VOG, symptom-onset-to-video-oculography; SN, spontaneous nystagmus; Sx-to-HIT, symptom-onset-to-head-impulse-testing; U, upbeat*.

Using fundus photography, contraversive OT was detected in all patients tested with unilateral INO (*n* = 14, missing data in two patients). At least one component of OTR was observed in all patients with unilateral INO. In bilateral INO, none of the five patients showed skew deviation or head tilt, even though fundus photography was not performed. On clinical examination or on quantitative VOG, spontaneous nystagmus was noted in 11 of 20 patients (missing data in one patient). This included dissociated vertical-torsional nystagmus (*n* = 8), downbeat nystagmus (*n* = 2), and upbeat nystagmus (*n* = 1) (Table 1). The pattern of dissociated vertical-torsional nystagmus was either ipsiversive torsional with a larger upbeat component in the contralesional eye (*n* = 6) or ipsiversive torsional in both eyes with vertical components in the opposite directions (*n* = 2).

### vHIT Findings

**Figure 2:**
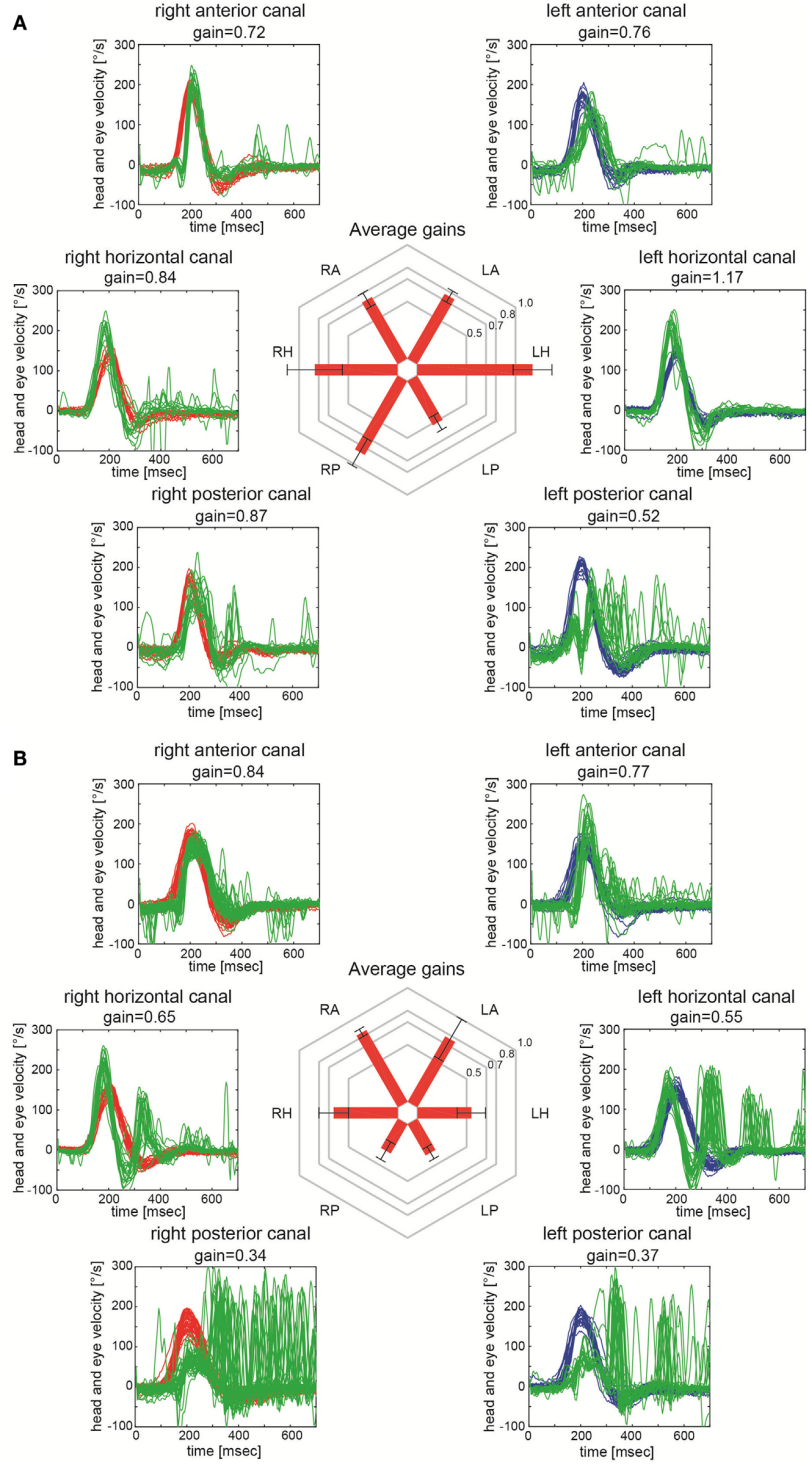
Video-head-impulse testing in two patients with internuclear ophthalmoplegia (INO). Eye velocity traces (in green) and head velocity traces (in red for testing the right vestibular organ and in blue for assessing the left vestibular organ) are plotted against time. Summary plots in the center illustrate average individual vestibulo-ocular reflex (VOR)-gains ± 1SD for all six canals. **(A)** Significant VOR-gain reduction accompanied by overt catch-up saccades in the contralesional (left) posterior semicircular canal (PSC) in a patient with right-sided INO (patient 7; see also MR-image in Figure 1A). Note that all other canals showed normal responses. **(B)** Significant VOR-gain reduction and catch-up saccades in both PSCs and both HSCs in a patient with bilateral INO (patient 19; see also MR-image in Figure 1B).

The time interval between symptom-onset and video-head-impulse testing in our patients varied (range = 1–1,072 days, Table 1). However, all patients had clinically and radiologically confirmed INO at the time of testing. Two illustrative cases for unilateral (Figure 2A) and bilateral (Figure 2B) INO on vHIT demonstrate involvement of the vertical semicircular canals, being most prominent for the (contralesional) PSC.

#### Analysis of VOR-Gains

When comparing the VOR-gains in the patients with unilateral INO vs. the healthy controls, statistical analysis (generalized linear model) indicated a main effect for the group and the different semicircular canals (Table 2). Furthermore, a significant interaction between the groups and the canals was noted. Pairwise comparisons demonstrated a characteristic pattern: in patients with unilateral INO (*n* = 16), VOR-gain reductions (when compared to controls) were most pronounced for the contralesional PSC (0.55 ± 0.11, *p* < 0.001). For both HSCs and ASCs and the ipsilesional PSC, we noted small but significant VOR-gain reductions as well (see Table 3). Compared to the contralesional ASC, VOR-gains in the contralesional PSC were significantly lower (*p* < 0.001), while this was not the case when comparing VOR-gains of the ipsilesional vertical canals (ASC vs. PSC, *p* = 0.658).

**Table 2:** main effects and interactions for aVOR gains (generalized linear model).

	Main effects						Interactions^a^	
	Groups		Semicircular canals		Side		Various	
Type of INO	Conditions	Statistics	Conditions	Statistics	Conditions	Statistics	Conditions	Statistics
Unilateral INO	Ipsilesional side INO patients, contralesional side INO patients, controls	df = 2	Horizontal, anterior, posterior	df = 2	NA	NA	Groups * canals	df = 4
		chi^2^ = 82.805		chi^2^ = 41.054				chi^2^ = 22.224
		*p* < 0.001		*p* < 0.001				*p* < 0.001

Right-sided unilateral INO	Right-sided INO patients, controls	df = 1	Horizontal, anterior, posterior	df = 2	Left, right	df = 1	Groups * sides * canals	df = 2
		chi^2^ = 78.334		chi^2^ = 40.463		chi^2^ = 0.038		chi^2^ = 16.691
		*p* < 0.001		*p* < 0.001		*p* = 0.846		*p* < 0.001

Left-sided unilateral INO	left-sided INO patients, controls	df = 1	Horizontal, anterior, posterior	df = 2	Left, right	df = 1	Groups * sides * canals	df = 2
		chi^2^ = 72.625		chi^2^ = 49.344		chi^2^ = 0.764		chi^2^ = 3.106
		*p* < 0.001		*p* < 0.001		*p* = 0.382		*p* = 0.212

Bilateral INO	Bilateral INO patients vs. controls	df = 1	Horizontal, anterior, posterior	df = 2	NA	NA	Groups * canals	df = 2
		chi^2^ = 84.439		chi^2^ = 42.309				chi^2^ = 8.207
		*p* < 0.001		*p* < 0.001				*p* = 0.017

*df, degrees of freedom; INO, internuclear ophthalmoplegia; NA, not applicable*.

*^a^Only selected interactions are shown here that allowed pairwise comparison of single conditions (e.g., left posterior canal in left-sided INO patients vs. left posterior canal in controls)*.

**Table 3:** Comparisons of mean VOR-gains in patients with INO (*n* = 21) and healthy controls (*n* = 42).

	Patients	Controls (*n* = 42)	*p*-Value*
**Bilateral INO (*n* = 5)**			

	Mean VOR-gains^a^	Mean VOR-gains^a^	

HSC	0.82 ± 0.32	1.04 ± 0.17	**<0.001**
ASC	0.58 ± 0.19	0.88 ± 0.09	**<0.001**
PSC	0.43 ± 0.11	0.89 ± 0.08	**<0.001**

**Unilateral INO (*n* = 16)**			

	Mean VOR-gains	Mean VOR-gains^a^	

HSC			
Ipsilesional	0.77 ± 0.27	1.04 ± 0.17	<0.001
Contralesional	0.90 ± 0.24	1.04 ± 0.17	**0.003**
ASC			
Ipsilesional	0.75 ± 0.17	0.88 ± 0.09	**0.006**
Contralesional	0.76 ± 0.19	0.88 ± 0.09	**0.013**
PSC			
Ipsilesional	0.73 ± 0.18	0.89 ± 0.08	**0.001**
Contralesional	0.55 ± 0.11	0.89 ± 0.08	**<0.001**

**Right-sided INO only (*n* = 10)**			

	Mean VOR-gains	Mean VOR-gains	

HSC			
Right	0.73 ± 0.32	1.07 ± 0.17	**<0.001**
Left	0.91 ± 0.28	1.01 ± 0.18	0.069
ASC			
Right	0.76 ± 0.17	0.84 ± 0.11	0.144
Left	0.81 ± 0.18	0.92 ± 0.11	0.041
PSC			
Right	0.74 ± 0.18	0.91 ± 0.08	**0.001**
Left	0.55 ± 0.11	0.88 ± 0.10	**<0.001**

**Left-sided INO only (*n* = 6)**			

	Mean VOR-gains	Mean VOR-gains	

HSC			
Right	0.89 ± 0.17	1.07 ± 0.17	**0.001**
Left	0.84 ± 0.15	1.01 ±0.18	**0.004**
ASC			
Right	0.68 ± 0.22	0.84 ±0.11	**0.008**
Left	0.73 ± 0.18	0.92 ± 0.11	**0.001**
PSC			
Right	0.57 ± 0.12	0.91 ± 0.08	<0.001
Left	0.71 ± 0.20	0.88 ± 0.10	0.003

*Significant p-values are in bold.

*^a^Values from corresponding left and right semicircular canals (e.g., left and right anterior canals) were pooled for comparison*.

*ASC, anterior semicircular canal; B-INO, bilateral internuclear ophthalmoplegia; HSC, horizontal semicircular canal; INO, internuclear ophthalmoplegia; L, left; PSC, posterior semicircular canal; R, right; U-INO, unilateral internuclear ophthalmoplegia; VOR, vestibulo-ocular reflex*.

Focusing on patients with right-sided INO, statistical analysis demonstrated main effects for the groups and the canals, but not for the side (Table 2). A significant interaction was found between all three model-parameters. Pairwise comparisons are illustrated in Table 3, indicating significantly lower VOR-gain values for the contralesional ASC and PSC and for the ipsilesional HSC compared to the controls, whereas gains were not different for the ipsilesional HSC and ASC. VOR-gains were significantly lower for the contralesional PSC than for the contralesional ASC (p < 0.001) and significantly lower for the contralesional PSC than for the ipsilesional PSC (*p* = 0.003).

For patients with left-sided INO, statistical analysis demonstrated main effects for the groups and the canals, but not for the side (Table 2). No significant interactions were found. Pairwise comparisons are illustrated in Table 3, indicating significantly lower VOR-gain values for all six canals in the patients. There was a trend for lower VOR-gains for the contralesional PSC than for the ipsilesional PSC (*p* = 0.070), while on the contralesional side no significant differences in VOR-gain for the ASC and the PSC were noted (*p* = 0.127).

In patients with bilateral INO (*n* = 5), statistical analysis indicated a main effect for the groups and the different semicircular canals. Furthermore, a significant interaction between the groups and the canals was noted (Table 2). Compared to controls, pairwise comparisons demonstrated significant VOR-gain reductions for the ASCs, HSCs and PSCs (*p* < 0.001), with a trend for lower VOR-gains for the PSCs than for the ASCs (0.43 ± 0.14 vs. 0.58 ± 0.19, *p* = 0.078). In comparison to the HSCs, VOR-gains in the vertical canals were significantly lower (*p* ≤ 0.002).

Comparing the VOR-gains of specific canals on the affected (ipsilesional) and the healthy (contralesional) side in patients with unilateral INO (*n* = 16), the VOR-gains of the contralesional PSC (*p* = 0.002) and the contralesional HSC (*p* = 0.016) were significantly smaller, while no significant differences (*p* > 0.05) were noted for the ASCs (Table 4).

**Table 4:** Comparison of VOR-gains of ipsilesional vs. contralesional semicircular canals in patients with unilateral internuclear ophthalmoplegia (*n* = 16).

	Ipsilesional	Contralesional	*p*-Value*
HSC	0.77 ± 0.27	0.90 ± 0.24	**0.016**
ASC	0.75 ± 0.17	0.76 ± 0.19	0.829
PSC	0.73 ± 0.18	0.55 ± 0.11	**0.002**

*Significant p-values are in bold.

*ASC, anterior semicircular canal; HSC, horizontal semicircular canal; PSC, posterior semicircular canal; VOR, vestibulo-ocular reflex*.

#### Overall Function of Individual Semicircular Canals and Presumed Diagnosis

In their overall rating, the reviewers correctly identified the INO-pattern (right-sided, left-sided, bilateral) in 16 out of 21 patients and judged all 42 healthy control subjects as normal, resulting in an overall sensitivity of 84.2% [95%-CI = 0.59.5–95.8] and a specificity of 95.5% [95%-CI = 83.3–99.2]. There was one patient with left-sided INO that was missed (false-negative) and two patients with bilateral INO that were rated as left-sided INO instead. In two patients false-positive findings were noted with bilateral INO instead of unilateral INO.

In cases rated as unilateral INO by the two reviewers, involvement of other canals besides the contralesional PSC was reported in 6/15 (40%) cases (note that one case with unilateral INO was rated as normal by the reviewers). Most frequently, involvement of the ipsilesional HSC was described (4/15), being accompanied by contralesional impairment of the HSC in two cases. Loss-of-function was reported in the ipsilesional (*n* = 2) and the contralesional (*n* = 2) ASC. In the five INO-cases rated as bilateral, involvement of either both ASCs or one HSC, respectively, were seen in two patients each.

#### Analysis of Compensatory Saccades

**Figure 3:**
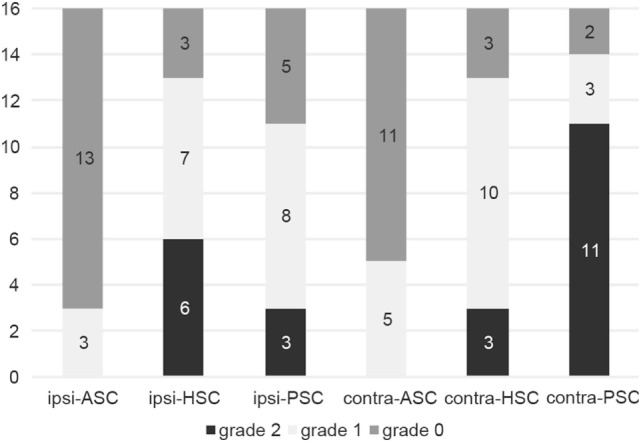
Frequency of compensatory catch-up saccades in patients with unilateral internuclear ophthalmoplegia (*n* = 16). For each semicircular canal the fraction of patients with either no catch-up saccades (grade 0, in dark gray), catch-up saccades in less than 50% of traces (grade 1, in light gray) and catch-up saccades in more than 50% of traces (grade 2, in black) is shown in a box plot. Abbreviations: Contra-ASC, contralesional anterior semicircular canal; Contra-HSC, contralesional horizontal semicircular canal; Contra-PSC, contralesional posterior semicircular canal; Ipsi-ASC, ipsilesional anterior semicircular canal; Ipsi-HSC, ipsilesional horizontal semicircular canal; Ipsi-PSC, ipsilesional posterior semicircular canal.

**Figure 4:**
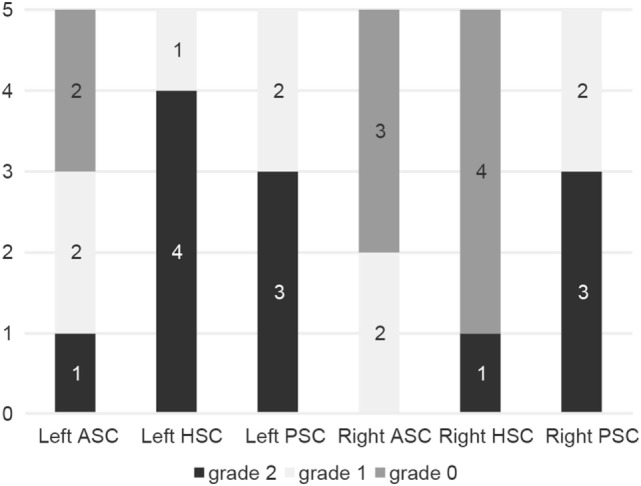
Frequency of compensatory catch-up saccades in patients with bilateral internuclear ophthalmoplegia (*n* = 5). For each semicircular canal the fraction of patients with either no catch-up saccades (grade 0, in dark gray), catch-up saccades in less than 50% of traces (grade 1, in light gray) and catch-up saccades in more than 50% of traces (grade 2, in black) is shown in a box plot. For explanation of abbreviations see figure legend of Figure 3.

Compensatory catch-up saccades were detected predominantly in the contralesional PSC in unilateral INO (Figure 3) and in both PSCs in bilateral INO (Figure 4). In patients with unilateral INO, grade 2 compensatory saccades were observed most commonly in the contralesional PSC (11/16, 69%), followed by the ipsilesional HSC (6/16, 37.5%). In patients with bilateral INO, grade 2 compensatory saccades were found commonly in the left HSC (4/5, 80%) and in both PSCs (3/5, 60%).

## Discussion

Unilateral INO is characterized by impaired adduction of the ipsilesional eye and dissociated nystagmus of the contralesional abducting eye due to a lesion involving the MLF that carries signals from the contralateral abducens nucleus to the ipsilateral medial rectus subnucleus of the oculomotor nuclear complex (2, 3, 20). In addition, the MLF is also known to transmit excitatory VOR-commands from the contralateral ASC and PSC to the ocular motor nuclei, and graviceptive input from the otolith organs and the vertical semicircular canals to the contralateral interstitial nucleus of Cajal (2, 7). Using video-head-impulse testing to assess the pattern of vertical semicircular canal function in unilateral and bilateral INO-patients, we observed predominant or even selective impairment of the PSCs, being contralesional in unilateral INO and bilateral in bilateral INO. To a lesser degree, involvement of the ASCs was noted as well.

### VOR-Abnormalities in INO

The MLF conveys VOR-signals from the contralateral vertical canals. In a previous study in a single patient with right-sided INO using binocular magnetic search coils, head-impulse testing for the left PSC demonstrated severly impaired VOR-responses in both eyes, while those for the left ASC were mildly reduced only (16). In unilateral INO, we noted a marked decrease in VOR-gain for the PSC, while the VOR-gain of the contralesional ASC was reduced slightly only (0.55 ± 0.11 vs. 0.76 ± 0.19, *p* < 0.001). Likewise, in bilateral INO, there was a trend to lower vertical VOR-gains for the PSCs than for the ASCs (*p* = 0.078).

Based on research in non-human primates and the single patient described above, it has been proposed that the VOR-signals from the PSC are transmitted exclusively through the MLF, whereas those from the ASC pass also through extra-MLF pathways such as the ventral tegmental tract (VTT) and the brachium conjuntivum (16, 21–23). Our observation that VOR-gains and overall canal function (as rated by two independent reviewers) were impaired the most in the contralesional PSC in unilateral INO and in both PSCs in bilateral INO is consistent with this hypothesis and further emphasizes the dissociation between PSC- and ASC-function in patients with INO.

Based on the ratings of semicircular canal function, involvement of other canals was found in 40% of unilateral INO-patients and in 80% of bilateral INO-patients. While involvement of the ASCs (contralesional in unilateral INO, bilateral in bilateral INO) can be explained by the projections from the ASC passing through the contralateral MLF, involvement of the ipsilesional ASC and PSC and of the HSCs need further discussion. Loss-of-function of the ipsilesional HSC may result from slow or absent adduction of the ipsilesional eye in patients with right-sided INO, as vHIT-measurements were always obtained from the right eye. Based on the overall rating of semicircular canal function, ipsilesional impairment of the ASC and/or PSC was noted in two patients with unilateral INO only, suggesting that this is an infrequent finding. In one of these two patients (patient 5), the vHIT was suggestive of bilateral INO, whereas on clinical evaluation right-sided INO was noted only, accompanied by a lesion including the left MLF on MRI. Such discrepancy could reflect more subtle (i.e., subclinical) involvement of the right MLF in this patient as well. Involvement of additional canals in part of the INO-patients as identified by the reviewers is also reflected by the VOR-gain values in the PSCs, ASCs, and HSCs. Whereas significant gain reductions (compared to the controls) were noted for the ipsilesional PSC and ASC and both HSCs in unilateral INO, the VOR-gain values were significantly (*p* < 0.001) larger than those for the contralesional PSC and often within normal range [i.e., VOR-gain ≥0.7 (vertical canals) and ≥0.8 (horizontal canals), respectively], suggesting minor involvement only. A possible explanation for loss-of-function of the ipsilesional PSC/contralesional HSC is compensatory VOR-gain downregulation in case of contralesional PSC/ipsilesional HSC involvement.

### Patterns of Catch-Up Saccades

Compensatory (“catch-up”) saccades are generated when the VOR is impaired, to maintain images stable on the fovea. The presence of catch-up saccades is the signature feature of VOR dysfunction and correlates with the VOR-gain deficit (17). In patients with unilateral INO, catch-up saccades were most commonly observed in the contralesional PSC, consistent with the most pronounced VOR-gain impairments for this canal. However, in both HSCs, a moderate degree of compensatory saccades was observed. The horizontal gaze palsy in the ipsilesional eye and the dissociated abducting nystagmus in the contralesional eye might be confounding factors in this context. In cases with bilateral INO, compensatory saccades were also most frequently found in the PSCs.

### Spontaneous Nystagmus in INO

Besides the characteristic dissociated abducting nystagmus, spontaneous nystagmus is often observed in INO-patients (5, 8, 24). In unilateral INO, dissociated vertical-torsional nystagmus (jerky seesaw nystagmus) may be present (8, 25). Dissociated vertical-torsional nystagmus in INO has been ascribed to the disruption of vertical-torsional VOR-pathways (from the contralateral ASC/PSC through the MLF to the ocular motor nuclei) or the graviceptive pathways. In our series, torsional nystagmus was ipsiversive as previously reported (5), and the upbeat component was larger in the contralesional eye. In a previous study analyzing distinct types of dissociated vertical-torsional nystagmus in unilateral INO, there were three major patterns of spontaneous nystagmus in INO (5). The most common nystagmus was ipsiversive torsional nystagmus with a larger upbeat component in the contralesional eye (55%); followed by ipsiversive torsional nystagmus in both eyes with vertical components in the opposite directions (33%) and ipsiversive torsional nystagmus with a larger downbeat component in the ipsilesional eye (12%) (5). In accordance with these results, we noted ipsiversive torsional nystagmus with a larger upbeat component in the contralesional eye (6/8, 75%) and ipsiversive torsional nystagmus in both eyes with vertical components in the opposite directions (2/8, 25%) in our series.

First, to explain the nystagmus caused by the lesion of the vertical VOR-pathway in unilateral INO, damage to the pathway in the MLF (from the contralesional ASC) would cause primarily a downward deviation of the contralesional eye and an intorsion of the ipsilesional eye (26). The resulting corrective nystagmus quick phases would be mostly upbeating in the contralesional eye and extorsional in the ipsilesional eye. However, from the perspective of VOR-gain, vHIT showed a decreased PSC-gain rather than the above-presumed mechanism. In our two patients with dissociated vertical-torsional nystagmus and INO, the torsional component of spontaneous nystagmus was in the opposite direction of OTR. These findings indicate that the dissociated vertical-torsional nystagmus may be a compensatory phenomenon of contraversive OTR and suggest an additional role of the graviceptive pathways originating from the otolith organs in the generation of dissociated vertical-torsional nystagmus in INO (5).

In bilateral INO, pure upbeat or downbeat nystagmus was observed. Pure upbeat nystagmus is explained by damage to the pathways from both ASCs due to bilateral MLF lesions. On the contrary, pure downbeat nystagmus may be caused by selective damage to the pathways from both PSCs. Why do similar MLF lesions sometimes cause dysfunction reflecting impairment of the ASC pathways and sometimes indicating impairment of the PSC pathways? From the perspective of VOR-gains, the direction of vertical spontaneous nystagmus could potentially be explained by the relative VOR-gain impairments: while VOR-gains of the ASCs were identical with those of the PSCs (ASC-gain = 0.58, PSC-gain = 0.58) in the subject that showed upbeat nystagmus (patient 18), VOR-gains of the PSCs were lower than those of the ASCs (PSC-gains = 0.40 and 0.35, ASC-gains = 0.62 and 0.84) in those subjects that presented with downbeat nystagmus (patients 17 and 19). Therefore, although the sample size was small, the direction of the vertical nystagmus in bilateral INO-cases may be related to the VOR-gain ratio of the vertical semicircular canals (ASC-gain/PSC-gain).

In a report on upbeat nystagmus in association with bilateral INO (27), another mechanism suggested was deficient neural-integrator function rather than an imbalance in the vertical VOR, since the slow-phase velocity of the vertical nystagmus was decreased and bedside HITs in the vertical plane were normal (27). However, in our series, the upbeat nystagmus had constant slow-phase velocities, and abnormal vHIT findings were more consistent with an imbalance in the vertical VOR.

In bilateral INO cases presenting with upbeat nystagmus, this could also be explained by concomitant bilateral lesions in the VTT (22), leading to hypoactivity of the elevator muscle motor neurons with respect to the unchanged downward system, eliciting downward eye-drift and upbeat nystagmus (28). In the single patient with bilateral INO and upbeat nystagmus, MRI demonstrated a more extensive lesion than in the other bilateral INO cases. However, whether the VTT was indeed affected in this patient, remains open.

### Differential Diagnosis of vHIT Findings in INO

Clinically, the distinction between peripheral-type and central-type acute vestibular syndrome (AVS) is important (12). With regards to impairment of one PSC, this can be seen both in patients with inferior vestibular neuritis (i.e., peripheral-type AVS) and in patients with INO (reflecting central-type AVS). Furthermore, both disease entities may present with torsional nystagmus with a downbeat component. INO-patients may show other patterns of spontaneous nystagmus and may have abnormal VOR-gains in other semicircular canals (5, 29). Of note, usually characteristic central eye movement abnormalities, such as ipsilesional adducting gaze impairment and dissociated abducting nystagmus in the contralesional eye can be seen in patients with INO. However, in some instances, especially when the medial gaze impairment is subtle or slowing of adduction is mild, the distinction between inferior vestibular neuritis and INO at the bedside can be very difficult. Obtaining video-head-impulse testing in these patients may help in the differential diagnosis (e.g., when documenting additional involvement of the ASC on the same side as consistent with INO) along with MR-imaging ordered.

Gold standard in the diagnosis of INO is MR-imaging, ordered because of a clinical suspicion of INO. In our study, we found an overall sensitivity of 84.2% and a specificity of 95.5% for the vHIT in diagnosing the pattern of INO. For MRI (combining FLAIR, proton-density and T2-weighted imaging), sensitivity and specificity values of 79.3 and 86.6% have been published (30). In a study by Frohman and colleagues, the accuracy of clinical detection of INO was assessed. Quantitative infrared oculography was used to grade the severity of adduction weakness and 279 physicians were shown video recordings of INO patients and controls. Detection rates depended on the degree of adduction weakness, being highest for cases with severe adduction deficits (94%) and lowest for mild adduction slowing (29%) (31). To which extent the vHIT improves the diagnostic accuracy of INO needs to be assessed prospectively in future studies in a head-to-head comparison with MRI and clinical examination.

### Limitations

Vertical eye movements in INO have been shown to be conjugate (16), allowing the use of monocular recording systems to assess their function. However, with the monocular vHIT recording system used in this study, any interpretation of HSC function must be made with caution as horizontal eye movements in INO are disconjugate. To address this limitation, we have assessed HSC function separately in patients with right-sided and left-sided unilateral INO (Table 2). For those patients with right-sided INO, significant VOR-gain reductions were noted only when testing the ipsilesional HSC (which requires an adduction of the right eye), but not for the left (contralesional) HSC, most likely reflecting gaze palsy rather than impairment of the HSC. For patients with left-sided INO, significant gain reductions were noted for both HSCs. Whether this reflects a pathophysiological process as part of the INO or this is an artifact remains open. Compared to the VOR-gain impairments of the ipsilesional HSC in right-sided INO-patients, these reductions were much smaller. Furthermore, vHIT ratings were reviewer-dependent and calculated sensitivity and specificity values yielded broad confidence intervals due to the relatively small sample size. Diagnostic accuracy may, therefore, have been overestimated.

## Conclusion

In patients with acute dizziness yielding abnormal HIT results, especially in the PSC plane, the differential diagnosis should include INO as a potential central cause. Using a monocular vHIT system, we could reliably evaluate vertical VOR function in patients with INO, confirming previously reported predominant impairment of the PSC opposite to the side of the INO. The more subtle impairment of overall canal function and VOR-gains and the compensatory catch-up saccade pattern in the ASCs compared to the PSCs, suggests that the ASC signal does not travel exclusively through the MLF and may pass through other pathways.

## Ethics Statement

This study was carried out in accordance with the recommendations of the Institutional Review Board of the Chonnam National University Hospital (Gwangju, South Korea) with written informed consent from all subjects. All subjects gave written informed consent in accordance with the Declaration of Helsinki. The protocol was approved by the Institutional Review Board of the Chonnam National University Hospital (Gwangju, South Korea).

## Author Contributions

SHL drafted the manuscript, performed the data collection, analyzed the data, and conceived of the study. SHK analyzed the data and conducted the statistical analysis. SSK performed the data collection, interpreted neuro-images, and analyzed the data. KK performed the data collection and analyzed the data. AT was involved in the design of the study, participated in the data analysis and the statistical analysis, and critically reviewed and edited the manuscript. All authors read, revised, and approved the final version of the manuscript.

## Conflict of Interest Statement

The authors declare that the research was conducted in the absence of any commercial or financial relationships that could be construed as a potential conflict of interest.
